# A Novel Optimized Silver Nitrate Staining Method for Visualizing and Quantifying the Osteocyte Lacuno-Canalicular System (LCS)

**DOI:** 10.21769/BioProtoc.5289

**Published:** 2025-04-20

**Authors:** Jinlian Wu, Chunchun Xue, Qiang Li, Hongjin Wu, Jie Zhang, Chenglong Wang, Weiwei Dai, Libo Wang

**Affiliations:** 1 Central Laboratory of Science and Technology, Longhua Hospital Shanghai University of Traditional Chinese Medicine, Shanghai, China; 2 Department of Rheumatology and Immunology, Longhua Hospital Shanghai University of Traditional Chinese Medicine, Shanghai, China; 3 Shanghai Municipal Hospital of Traditional Chinese Medicine, Shanghai University of Traditional Chinese Medicine, Shanghai, China; 4 Longhua Hospital Shanghai University of Traditional Chinese Medicine, Shanghai, China

**Keywords:** Silver Nitrate, Gelatin, Formic acid, Osteocyte, Lacuno-canalicular System (LCS), Bone

## Abstract

The osteocyte lacuno-canalicular system (LCS) plays a crucial role in maintaining bone homeostasis and mediating cellular mechanotransduction. Current histological techniques, particularly the Ploton silver nitrate staining method, face challenges such as variations in solution concentrations and types as well as a lack of standardization, which limits their broader application in osteocyte research. In this study, we present a simplified and more effective silver nitrate staining protocol designed to address these issues. Our method utilizes a 1 mol/L silver nitrate solution combined with optimized gelatin-formic acid solutions at varying concentrations (0.05%–0.5% type-B gelatin and 0.05%–5% formic acid, or 1%–2% type-B gelatin and 0.1%–2% formic acid). Staining is performed for 1 h under 254 nm ultraviolet light or 90 min under room light, followed by washing with Milli-Q water to terminate staining. This novel optimized method yields consistent and distinct staining of the osteocyte LCS across multiple species, demonstrating superior efficiency and reliability compared to the Ploton method. It will significantly advance research in osteocyte biology and provide a valuable tool for exploring the adaptive evolution of osteocyte LCS morphology and function across various taxa.

Key features

• A novel optimized silver nitrate method using a 1 mol/L silver nitrate solution with type-B gelatin-formic acid solution effectively stains the osteocyte LCS.

• The novel optimized method is more efficient for staining the osteocyte LCS across different species.

• The novel optimized method is simpler to perform and more cost-effective than conventional methods.

Graphical overview

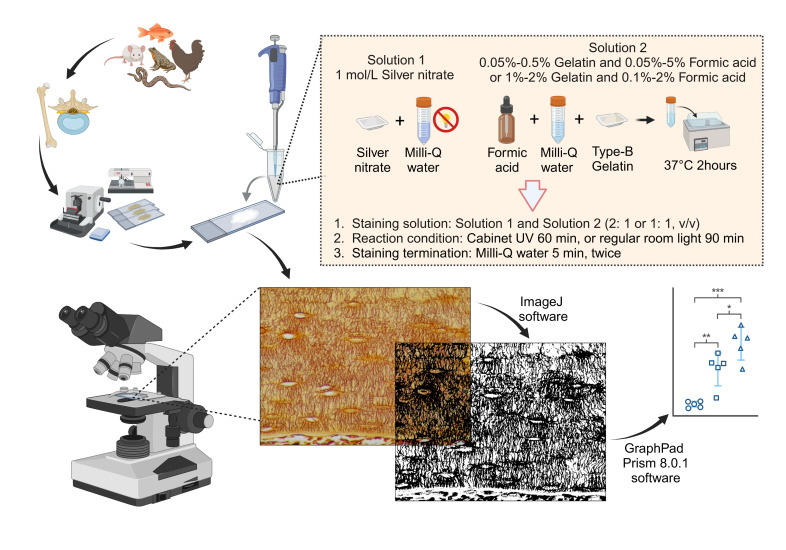

**Overview of the silver nitrate staining method for staining and qualifying osteocyte lacuno-canalicular system (LCS).** Created in BioRender. Libo, W. (2024) https://BioRender.com/c10z002

## Background

Osteocytes and their lacuno-canalicular system (LCS) are crucial for maintaining bone homeostasis and regulating cellular mechanotransduction. The LCS facilitates nutrient exchange, waste removal, and cell signaling between osteocytes, osteoblasts, osteoclasts, and vascular endothelial cells, thereby playing a significant role in bone development and health [1–4].

The characterization and quantification of the osteocyte lacuno-canalicular system (LCS) have primarily relied on impregnation staining techniques using high concentrations of silver nitrate, particularly the 50% solution introduced by Goodpasture and Bloom in 1975 [5]. This method was adapted from earlier studies, including the work by Howell et al. on staining chromosomal nucleolus organizer regions using a 10% silver nitrate solution [6]. In 1980, Howell and Black simplified the staining procedure, developing a one-step method that improved reproducibility and ease of use [7]. In 1986, Ploton et al. modified this method by lowering the reaction temperature from 70 to 20 °C and introducing a 5% sodium thiosulfate solution to terminate the staining process, thereby enhancing the technique's overall efficiency [8]. Later, Chappard et al. applied Ploton’s method to stain nucleolus organizer regions in undecalcified, methyl methacrylate (MMA)-embedded bones, inadvertently discovering its effectiveness for staining the osteocyte LCS [9–10]. They also established optimal staining conditions as 55 min at room temperature in the dark, which have since become the standard method [11–12].

By the early 2000s, the importance of osteocytes and LCS in bone remodeling had gained widespread attention. Researchers began adopting these staining solutions and protocols to study osteocyte biology. This method, commonly referred to as the Ploton silver nitrate staining method, facilitated significant advancements in understanding the role of osteocytes in bone health and disease [13–16]. Despite its widespread use, the Ploton method is influenced by various factors—such as silver nitrate concentration, gelatin-formic acid concentration, gelatin type, lighting conditions, and termination methods—that can result in unclear staining backgrounds, as shown in some studies [17–18]. These variables can hinder the accurate quantitative analysis of the osteocyte LCS.

In this paper, we present a novel optimized method that uses a 1 mol/L silver nitrate solution combined with a gelatin-formic acid solution at varying concentrations (0.05%–0.5% type-B gelatin and 0.05%–5% formic acid, or 1%–2% type-B gelatin and 0.1%–2% formic acid). The staining process is carried out under 254 nm ultraviolet light for 60 min or under regular room light for 90 min, followed by termination of the reaction with Milli-Q water. This novel optimized method enhances the efficiency of the staining process and effectively stains the osteocyte LCS across different vertebrate taxa. It offers a valuable tool for studies of osteocyte biology and LCS morphogenesis, providing new insights into the role of osteocytes in bone biology.

## Materials and reagents

**Biological materials**

1. 8-week-old male C57BL/6J mice and 8-week-old Sprague-Dawley rats were obtained from Hangzhou Ziyuan Laboratory Animal Technology Co., Ltd., Hangzhou, China

2. Rabbit (*Oryctolagus cuniculus*), silver carp (*Hypophthalmichthys sp.*), common carp (*Cyprinus carpio*), Tai Lake white fish (*Anabarilius sp.*), bullfrogs (*Lithobates catesbeiana*), chickens (*Gallus gallus domesticus*), and wall lizards (*Gekko sp.*) were obtained from a local food market and an online traditional Chinese medicine herb store

**Reagents**

1. 10% EDTA decalcifying solution (Sangon Biotech, catalog number: E671001–0500)

2. Sodium chloride (Sangon Biotech, catalog number: A610476)

3. Potassium chloride (Sangon Biotech, catalog number: A610440)

4. Sodium phosphate dibasic (Sangon Biotech, catalog number: A501727)

5. Potassium dihydrogen phosphate (Sangon Biotech, catalog number: A600445)

6. Silver nitrate (Sangon Biotech, catalog number: C510027–0010)

7. 10% neutral buffered formalin (Wexis, catalog number: 311010014)

8. Paraffin (Leica, catalog number: 39601006)

9. Ethanol (Sinopharm, catalog number: 10009218)

10. Xylene (Sinopharm, catalog number: 10023418)

11. 88% formic acid (Sinopharm, catalog number: 10010118)

12. Type-B gelatin (Solarbio, catalog number: G8061)

13. Neutral balsam mounting medium (Solarbio, catalog number: G8590)

14. Cedar oil (Sangon Biotech, catalog number: A502219–0025)

**Solutions**

1. 1 mol/L silver nitrate solution (see Recipes)

2. 1% (v/v) formic acid solution (see Recipes)

3. 2% (w/v) type-B gelatin in 1% formic acid solution (see Recipes)

4. Staining solution (see Recipes)

5. PBS solution (0.01 mol/L, pH 7.4) (see Recipes)

**Recipes**

**1. 1 mol/L silver nitrate solution**

**Table:** 

Reagent	Final concentration	Quantity or Volume
Silver nitrate powder	1 mol/L	8.75 g
Milli-Q water (18.2 MΩ·cm)	n/a	50 mL
Total	n/a	50 mL

Prepare the solution and store it at room temperature, protected from light. You can adjust the volume up or down depending on the number of slides to be stained. You can also purchase the pre-prepared 1 mol/L silver nitrate solution online, which is readily available and commonly used in chemical titration experiments.

**2. 1% (v/v) formic acid solution**

**Table:** 

Reagent	Final concentration	Quantity or Volume
Formic acid (88%)	1% (v/v)	0.568 mL
Milli-Q water (18.2 MΩ·cm)	n/a	49.432 mL
Total	n/a	50 mL

Store the solution at room temperature. You can adjust the volume up or down depending on the number of slides to be stained.

**3. 2% (w/v) type-B gelatin in 1% formic acid solution**

**Table:** 

Reagent	Final concentration	Quantity or Volume
Type-B gelatin	0.02 g/mL	1 g
1% formic acid solution	n/a	50 mL
Total	n/a	50 mL

Dissolve the mixture by warming it in a 37 °C water bath for approximately 2 h. Once the mixture has fully dissolved, it can be stored at room temperature. You can adjust the volume up or down depending on the number of slides to be stained. Based on our experience, a gelatin-formic acid solution can be prepared using either 0.05%–1% (w/v) type-B gelatin and 0.05%–5% (v/v) formic acid or 1%–2% (w/v) type-B gelatin and 0.1%–2% (v/v) formic acid. This formulation yields staining results as clear and effective as those obtained with a 2% (w/v) type-B gelatin in 1% formic acid solution.

**4. Staining solution**

**Table:** 

Reagent	Final concentration	Quantity or Volume
1 mol/L silver nitrate solution	2/3 or 33.3% (v/v)	0.2 mL
2% type-B gelatin in 1% formic acid solution	1/3 or 66.7% (v/v)	0.1 mL
Total	n/a	0.3 mL

Prepare the staining solution immediately before use, ensuring it is kept away from light. This should be done after washing the tissue sections with Milli-Q water and applying the solution to the bone tissue on the slide without delay. Avoid preparing the solution in advance, as this may lead to poor or incomplete staining results. The volume ratio of silver nitrate solution to 2% gelatin and 1% formic acid solution can be either 2:1 or 1:1. Based on our experience, 0.3 mL of staining solution is sufficient for thorough staining of a slide with a single bone tissue section attached.

**5. PBS solution (0.01 mol/L, pH 7.4)**

**Table:** 

Reagent	Final concentration	Quantity or Volume
Sodium chloride	0.137 mol/L	8.006 g
Potassium chloride	2.7 mmol/L	0.201 g
Sodium phosphate dibasic	1.8 mmol/L	1.419 g
Potassium dihydrogen phosphate	0.01 mol/L	0.244 g
Milli-Q water (18.2 MΩ·cm)	n/a	1,000 mL
Total	n/a	1,000 mL

**Laboratory supplies**

1. 1.5 mL microcentrifuge tubes (Axygen, catalog number: MCT-150-C)

2. Microscope slides (Citotest, catalog number: 80312–3161)

3. Microscope cover slides (Citotest, catalog number: 80340–0130)

4. Pipettes (Gilson, model: P1000 and P200)

5. 50 mL tubes (Greiner, catalog number:227651)

6. Embedding cassettes (Citotest, catalog number: 31050102W)

7. Forceps and scissors

## Equipment

1. Biological safety cabinet (Thermo Fisher Scientific, model: 1379/1389 with 254 nm UV light G3675L 31~40 W)

2. Automatic benchtop tissue processor (Leica, model: TP1020)

3. Tissue embedding station (Leica, model: EG1160)

4. Rotary microtomes (Leica, model: RM2135)

5. Histology water bath (Leica, model: Histobath HI1210)

6. Ultrapure water purification system (Millipore, model: Milli-Q Elix^®^ Essential)

7. Water bath (JingHong, model: DNB-501S)

8. Thermoelectric oven (JingHong, model: DNP-9052)

9. Analytical balance (Sartorius, model: BSA124S)

10. Nikon brightfield microscope (Nikon, model: ECLIPSE 200)

## Software and datasets

1. ImageJ software (NIH, USA)

2. GraphPad Prism 8.0.1

3. BioRender (https://www.biorender.com/)

## Procedure

**A. Bone sample collection, embedding, and sectioning**

1. Euthanize 8-week-old male C57BL/6J mice and Sprague-Dawley rats by carbon dioxide inhalation. Then, briefly spray the mouse's skin with a 75% alcohol solution for general disinfection.

2. Perform a dissection to obtain the desired bone(s) (e.g., femur, tibia, or other bones of interest) using sterile scissors and forceps. Carefully remove any excess muscle tissue with the scissors and forceps and rinse the bone in cold PBS to remove blood and debris.

3. Transfer the bones to a fixative solution, such as 10% neutral buffered formalin or 4% PFA, which are commonly used and sufficient for most bone tissues. Ensure that the volume of the fixative solution is ten times that of the bones to ensure complete fixation. Fix the bones for 24 h at 4 °C.

4. After fixation, rinse the bones thoroughly in Milli-Q water six times, with each rinse lasting 10 min, to remove excess fixative.

5. Transfer the bones to a 10% EDTA (pH 7.4) decalcifying solution, changing the solution every 3 days to ensure complete decalcification. Monitor the progress by gently bending the bones weekly to check for hardness. The bones should become soft when fully decalcified. Once they become flexible, decalcification is complete. For complete long bones (tibia or femur) and lumbar vertebrae of C57BL/6J mice aged 8 weeks or older, as well as other skeletal samples of comparable size, a typical decalcification using 10% EDTA (pH 7.4) for four weeks is sufficient.

6. After decalcification, rinse the bone thoroughly in Milli-Q water six times, with each rinse lasting 10 min, to remove the decalcifying agent.

7. Dehydrate the bones through a graded ethanol series, sequentially immersing them in 70%, 80%, and 90% ethanol for 1 h each, followed by three rinses in 100% ethanol, with each rinse lasting 1 h to ensure complete dehydration.

8. Clear the bones in a graded xylene series: 50% ethanol–50% xylene (v/v), followed by 100% xylene twice, for 1 h in each solution.

9. Infiltrate the bone with molten paraffin at 60 °C. Repeat the process two more times for a total of three changes of molten paraffin to ensure full infiltration of the tissue.

10. Embed the bone in paraffin by transferring it to embedding molds filled with molten paraffin. Allow the paraffin to solidify at room temperature for approximately 1 h.

11. Once the paraffin in the embedding molds has fully solidified, place the molds in a -20 °C freezer for 10 min. After 10 min, remove the molds and gently detach the paraffin blocks containing bone tissues. The paraffin blocks can be stored long-term at room temperature or in a 4 °C refrigerator for at least three years.

12. Cut the embedded bone paraffin blocks into 4 μm thick sections using a microtome. Transfer the sections onto microscope slides, ensuring they are evenly distributed and properly adhered.

13. Dry the sections in a thermoelectric oven at 37 °C for 1 h to ensure good adherence to the slides.

14. Dewax the sections in a thermoelectric oven at 60 °C for 6 h to ensure complete removal of the surrounding wax and any residual moisture from the sections.

15. Store dewaxed sections at room temperature or 4 °C for long-term preservation for at least three years.

**B. Silver nitrate staining of osteocyte LCS**

1. Deparaffinization: Place the 4 μm bone sections in xylene for 10 min at room temperature. Repeat the process two more times for a total of three changes of xylene, ensuring complete removal of paraffin.

2. Hydration: Sequentially rehydrate the sections through a graded ethanol series: 100%, 90%, 80%, and 70% ethanol, for 5 min in each solution.

3. Washing: Wash the sections twice with 18.2 MΩ·cm Milli-Q water for 5 min each time to eliminate residual ethanol. Avoid using tap water, PBS, TBS, or saline-based solutions as they may interfere with the staining process.

4. Staining: After rehydrating the tissue sections, gently blot the surrounding area on the slide with absorbent paper, taking care not to touch the tissue itself to avoid distortion or loss of tissue integrity. Once excess water is removed, place the slides flat in a staining box to ensure even coverage of the tissue and prevent spillage of the staining solution. Prepare the staining solution according to Recipe 4 and immediately apply it to the bone tissue sections. Incubate the slides either under 254 nm UV light in a biological safety cabinet for 60 min or under regular room light for 90 min at room temperature.

5. Staining termination: Wash the slides twice with 18.2 MΩ·cm Milli-Q water for 5 min each time to remove excess staining solution. Avoid tap water, PBS, TBS, or saline-based solutions to prevent precipitation and ensure optimal staining quality.

6. Dehydration: Sequentially dehydrate the sections through a graded ethanol series: 70%, 80%, 90%, and 100%, for 5 min in each solution.

7. Clearing: Clear the sections by immersing the slides in xylene three times, for 10 min each, to eliminate ethanol and prepare for mounting.

8. Mounting: After clearing, apply a neutral balsam mounting medium to the sections and carefully cover the tissue with a coverslip. Ensure that no air bubbles are trapped between the slide and the coverslip.

9. Drying: Dry the mounted slides in a thermoelectric oven at 37 °C overnight. This will ensure that the neutral balsam solidifies completely and the tissue is properly embedded for further analysis.

**C. Imaging of osteocyte LCS**

**Figure 1.**
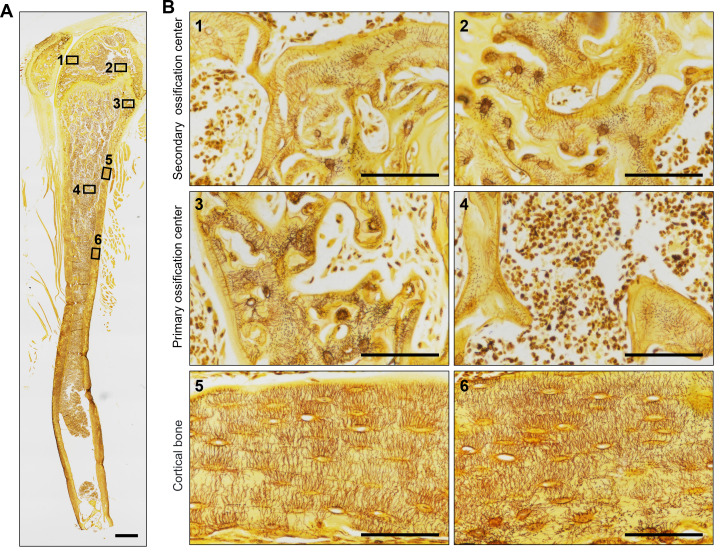
Staining effect of the new silver nitrate staining method on the tibia of mice. (A) Overall staining of the osteocyte lacuno-canalicular system (LCS) in the tibia of mice. The red box indicates the trabecular bone in the secondary ossification center, the green box indicates the trabecular bone in the primary ossification center, and the blue box indicates the cortical bone region. ×40 objective. Scale bar: 500 μm. (B) Magnified image showing the staining effect of the osteocyte LCS in three different regions of the tibia. Scale bars: 100 μm.

1. Use a high-resolution optical microscope with a high-magnification objective (×40 or ×100 in oil immersion) to capture osteocyte LCS. Acquire at least 4–5 images from randomly selected regions of the stained bone sections, ensuring the images represent areas that account for potential heterogeneity in bone structure. Maintain consistent lighting and exposure settings during image capture to ensure uniformity across all samples. As shown in Figure 1, the novel optimized silver nitrate method effectively stains the osteocyte LCS in the mouse tibia, producing clear staining in both trabecular and cortical bone.

2. Save the images in a TIF format with RGB color mode for further analysis.

## Data analysis

**Figure 2.**
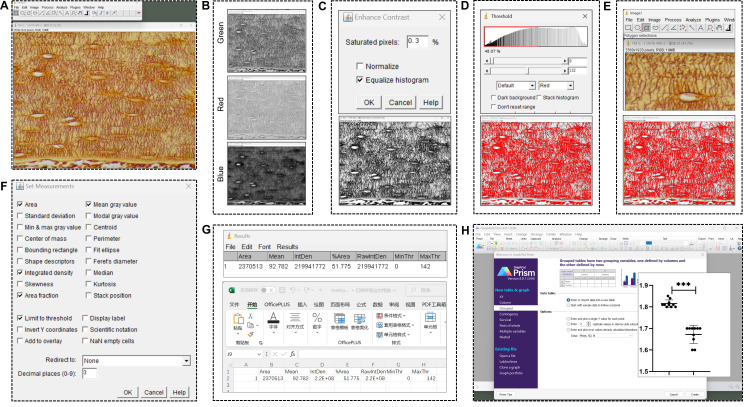
Processing and statistical analysis of novel optimized silver nitrate-stained osteocyte lacuno-canalicular system (LCS) images. (A) Open the TIF image in ImageJ software. (B) Split the images into the *red, green*, and *blue* channels, and use the *green* channel image for the subsequent steps. (C) Enhance the image contrast. (D) Set a threshold to distinguish positive LCS staining from the background. (E) Outline the region of interest (ROI) for measurement. (F) Measure the positive staining area within the ROI. (G) Export results. (H) Use GraphPad Prism 8.0.1 to perform comparisons and generate statistical plots.

Process and statistically analyze the osteocyte LCS images after silver nitrate staining as follows (Figure 2):

1. Open the TIF image (Figure 2A): Open the image in ImageJ by going to *Image > Open*.

2. Split the channels (Figure 2B): For an RGB image, split it into individual channels by selecting *Image > Color > Split Channels*. This will generate three separate images corresponding to the *red, green*, and *blue* channels. Select the green channel image (*green*) for the next steps.

3. Enhance image contrast (Figure 2C): To enhance contrast, go to *Process > Enhance Contrast*. Set the *Saturated pixels* to *0.3%* (the default setting in ImageJ) and select *Equalize histogram*. This step adjusts the image contrast using the software’s default settings.

4. Adjust brightness and contrast (optional): To further enhance visibility, adjust the brightness and contrast by selecting *Image > Adjust > Brightness/Contrast*. However, in this protocol, no adjustments were made to brightness and contrast in any of the images. To eliminate any variations due to manual adjustments, all images in this study used the software’s default settings without manual intervention.

5. Set threshold (Figure 2D): Set a threshold to distinguish positive LCS staining from the background by going to *Image > Adjust > Threshold*. Adjust the threshold visually until the positive staining areas closely match the original image. ImageJ will automatically select a default threshold value. To eliminate any variations due to manual adjustments, all images in this study used the default threshold value without manual intervention.

6. Define the region of interest (ROI) (Figure 2E): Use the *Polygon* or *Freehand* selection tools to outline the ROI for measurement.

7. Measure the ROI (Figure 2F): To analyze the selected area, go to *Analyze > Set Measurements*. Select the following parameters: *Area, Integrated density, Area fraction, Mean gray value*, and *Limit to threshold*. After setting these parameters, go to *Analyze > Measure* to measure the positive staining area. This will provide data on *Area, Integrated density, Area fraction*, and *Mean gray value*.

8. Repeat for other images or ROIs: Repeat the measurement process for each image or ROI.

9. Export results (Figure 2G): Copy the data from the *Results* window directly into Microsoft Excel or export it as a CSV file by selecting *Results > File > Save As*.

10. Statistics and graphs (Figure 2H): Comparisons of the normalized data between groups were made using either one-way ANOVA or an unpaired two-tailed Student's t-test, and statistical plots were generated using GraphPad Prism 8.0.1 software. Data were presented as means ± standard deviation, shown by error bars. A p-value < 0.05 was considered statistically significant.

## Validation of protocol

**Figure 3.**
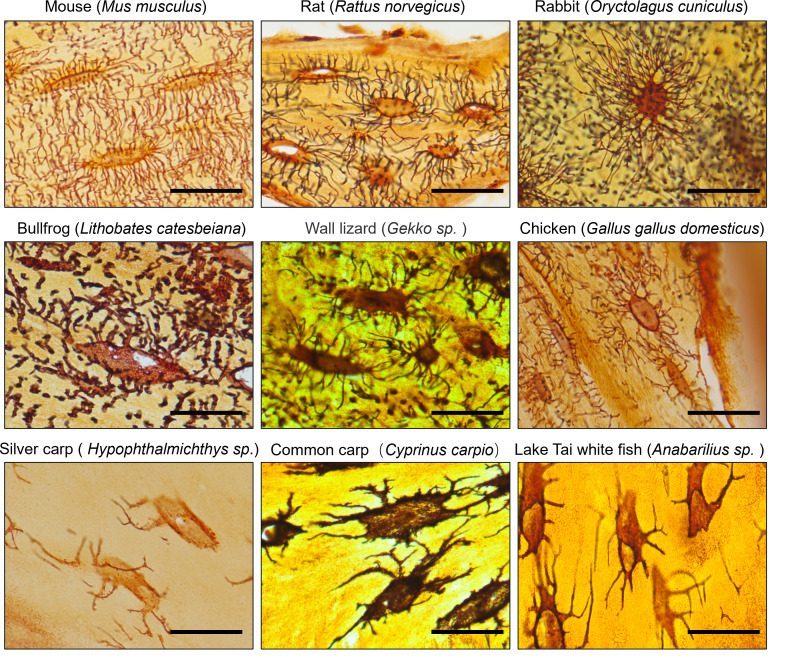
The novel optimized silver nitrate method effectively stains osteocyte lacuno-canalicular system (LCS) in various species. ×100 objective in oil immersion. Scale bars: 25 μm.

This protocol has been used and validated to stain osteocyte LCS across different species, consistently yielding clear and reliable staining results (Figure 3) in mammals (e.g., mouse, rat, rabbit), amphibians (e.g., bullfrog), reptiles (e.g., gecko), birds (e.g., chicken), and fish (e.g., silver carp, common carp, Tai Lake white fish).

This protocol is a crucial method in our ongoing research to identify phenotypes related to the role of alkaline phosphatase in a glucocorticoid-induced bone growth retardation mouse model, as well as the role of the Zeb1 transcription factor in a transgenic mouse model (Prrx1Cre;Zeb1^fx/fx^). This model exhibits pathological features similar to osteogenesis imperfecta and specifically overexpresses Zeb1 in skeletal mesenchymal stem cells. In these studies, we have adhered to a standardized methodology for characterizing and quantifying osteocyte LCS, obtaining consistent and high-quality staining results, as outlined in this protocol. This protocol has been used and validated in the following research article:

- Wu et al. [19]. A Novel Optimized Silver Nitrate Staining Method for Visualizing the Osteocyte Lacuno-Canalicular System. bioRxiv.

## General notes and troubleshooting

**General notes**

1. Storage and stability of gelatin-formic acid solution: The gelatin-formic acid solution can be stored at room temperature for several months but may undergo chemical changes over time. For example, a 2% gelatin–1% formic acid solution stored for 6 months requires a longer staining period (e.g., 2–3 h) to achieve the same level of LCS staining compared to a freshly prepared solution. Therefore, we recommend using freshly prepared gelatin-formic acid solutions within 3 months. If using a solution stored for longer than 3 months, you may need to extend the staining time to obtain optimal results.

2. Using 18.2 MΩ·cm Milli-Q water during staining: Tap water, PBS, TBS, or any solution containing salt will react with silver nitrate, forming a white flocculent precipitate. Therefore, slides should be rinsed with Milli-Q water after rehydration and staining to prevent any interference with the staining quality.

3. Handling of staining solution: The freshly prepared staining solution (a mixture of silver nitrate and gelatin-formic acid) is initially clear but will turn yellow within 5–10 min. We have found that yellow staining solutions are much less effective for LCS staining. Therefore, we recommend applying the freshly prepared solution to the slides within 5 min (or as quickly as possible).

4. Handling of slides: Slides can be handled directly with gloves throughout the procedure without compromising the quality of the staining. There is no need to use non-metallic tweezers or tools when handling slides during the staining process.

**Troubleshooting**

Problem 1: A large number of black particle precipitates appear on the glass slides and bone tissue areas after staining.

Solutions: (1) Check the preparation process of the gelatin-formic acid solution to ensure that gelatin was properly added. If gelatin was forgotten or if it has expired or deteriorated, it may result in a large amount of black particle precipitates on the stained slides, rendering the staining data unusable. (2) Use type-B gelatin. You may have used type-A gelatin or another inappropriate type for preparing the staining solution. Type-A gelatin can cause the formation of numerous black silver particle precipitates on the stained slides. Although the osteocyte LCS structure can still be clearly stained, the large amount of black particles adhering to the tissue cannot be easily removed by simple washing, rendering the staining data unusable.

Problem 2: After staining for 1 h under UV light in the cabinet, the number of stained osteocyte LCS is low.

Solutions: (1) Prepare a new gelatin-formic acid solution, ensuring that the solution is used within 3 months. The staining effect of gelatin-formic acid solutions decreases over time, so solutions stored for too long will yield poor results. (2) Check if the silver nitrate solution has turned yellow. Yellowed silver nitrate solutions result in poorer staining effects. If the solution has turned yellow, prepare a new silver nitrate staining solution and make sure to store it in the dark. (3) Extend the staining time. If you must use a gelatin-formic acid solution older than 3 months or a yellowed silver nitrate solution, you will need to extend the staining time, such as staining for 2–3 h. This can significantly improve the staining results and achieve clearer staining.
